# Spatial Distributions of Red Blood Cells Significantly Alter Local Haemodynamics

**DOI:** 10.1371/journal.pone.0100473

**Published:** 2014-06-20

**Authors:** Joseph M. Sherwood, David Holmes, Efstathios Kaliviotis, Stavroula Balabani

**Affiliations:** 1 Department of Bioengineering, Imperial College London, London, United Kingdom; 2 Department of Mechanical Engineering, University College London, London, United Kingdom; 3 London Centre for Nanotechnology, University College London, London, United Kingdom; 4 Sphere Fluidics Limited, The Jonas Webb Building, Babraham Research Campus, Babraham, Cambridge, United Kingdom; University of California San Diego, United States of America

## Abstract

Although bulk changes in red blood cell concentration between vessels have been well characterised, local distributions are generally overlooked. Red blood cells aggregate, deform and migrate within vessels, forming heterogeneous distributions which have considerable effect on local haemodynamics. The present study reports data on the local distribution of human red blood cells in a sequentially bifurcating microchannel, representing the branching geometry of the microvasculature. Imaging methodologies with simple extrapolations are used to infer three dimensional, time-averaged velocity and haematocrit distributions under a range of flow conditions. Strong correlation between the bluntness of the velocity and haematocrit profiles in the parent branch of the geometry is observed and red blood cell aggregation has a notable effect on the observed trends. The two branches of the first bifurcation show similar characteristics in terms of the shapes of the profiles and the extent of plasma skimming, despite the difference in geometric configuration. In the second bifurcation, considerable asymmetry between the branches in the plasma skimming relationship is observed, and elucidated by considering individual haematocrit profiles. The results of the study highlight the importance of considering local haematocrit distributions in the analysis of blood flow and could lead to more accurate computational models of blood flow in microvascular networks. The experimental approaches developed in this work provide a foundation for further examining the characteristics of microhaemodynamics.

## Introduction

Understanding and predicting blood characteristics is an important part of both clinical care and research into fundamental understanding of physiology and pathology. Chemical analyses of blood samples, in conjunction with several simple haemorheological assays, are widely used in diagnosis and monitoring of patients with a multitude of conditions. In order to understand the relevance of such tests, and to better understand disease states, it is important to fully appreciate the fundamental aspects of blood flow.

From a mechanical point of view, blood is essentially a multiphase fluid comprising red blood cells (RBCs) in plasma, a continuous Newtonian fluid. RBCs consist of a high viscosity fluid (relative to the plasma), surrounded by a membrane which can deform with ease, but strongly resists expansion [1]. The deformability of RBCs results in several phenomena which significantly affect haemodynamics. Due to their deformability, RBCs migrate away from vessel walls, resulting in a region of reduced RBC concentration (haematocrit) close to the walls. This leads to the Fåhraeus effect; a reduction in bulk haematocrit as vessel size decreases. In the large arteries, physiological haematocrit is 0.45, which reduces to as little as 0.1 in capillaries [2]. The region of low haematocrit near the vessel wall is commonly considered to be a cell-free or cell-depleted layer (CDL); the simplest assumption being that there is a geometrical region of a vessel through which no RBCs pass, and the haematocrit is constant elsewhere. Using high-speed photography, it is possible to measure the distance between the vessel wall and the first RBC optically, and thus to describe the CDL distribution in straight vessel sections. The influence of a number of parameters on CDL characteristics has been investigated [3]–[5]. A few recent studies in bifurcating geometries have highlighted the different CDL characteristics which arise in the branching environment of the vasculature [6], [7]. However, the description of the probabilistic properties of the CDL, such as mean width and roughness (with the assumption of a uniform haematocrit region), does not provide all of the relevant information. The distribution of RBCs in a vessel or channel varies continuously in the radial direction and has been measured by a number of researchers *in vitro* in straight geometries [6], [8], [9]. Cokelet et al. [10] provided detailed information on the haematocrit and velocity distributions in a Couette viscometer, and their results highlighted the significant shear and haematocrit dependence of haemodynamics. The shape of haematocrit distributions in bifurcating geometries has been described qualitatively [1], and *in vivo* in a section of an arteriolar network [11], but detailed parametric quantitative descriptions are still required. Recently, we quantified the haematocrit distribution in a simple T-bifurcation and showed that in the daughter branch the haematocrit distribution was asymmetric, and the velocity profiles correspondingly skewed in the opposite direction, due to the influence of local haematocrit on viscosity [12].

Microvessels regularly bifurcate, and the non-uniform haematocrit in the parent branch of bifurcations leads to a phenomenon known as ‘plasma skimming’, in which a disproportionally large number of cells enter the branch with the highest flow rate. A number of *in vitro* studies reported on the importance of various parameters by considering bulk values, i.e. average values for a given branch [13]–[16]. Fenton et al. [16] concluded that the most important parameters involved in plasma skimming were the parent branch haematocrit, the diameter of the channel and the flow ratio (the proportion of parent flow entering each side branch). The low radial dispersivity of RBCs (∼10^−7^ mm^2^/s [17]) means that between sequential bifurcations, the haematocrit profile, distorted by the flow field at the first bifurcation, may not have sufficient time/space to regain symmetry before the next bifurcation. Carr and Wickham [18] investigated serial bifurcations *in vitro* and concluded that the key parameter, aside from flow ratio, was the ratio of the distance between bifurcations to the flow rate. For larger values of this parameter, symmetry is recovered and the effect of the previous bifurcation is reduced. Carr and Xiao [19] modelled sequential bifurcations, taking into account the shift in RBC concentration and reported that approximately 40% of bifurcations in their simulation were affected by previous bifurcations. The distance between bifurcations is dependent on the specific microvascular network being considered; Bishop et al. [20] reported an average of 3.5∶1, while larger values of 8∶1 and 30∶1 have been reported elsewhere [21]. Pries et al. [22] carried out a comprehensive *in vivo* study of plasma skimming, and found that a length between bifurcations of approximately 10 was sufficient for the haematocrit profile to recover symmetry. A number of mathematical analyses of plasma skimming based on local RBC distribution have been given [14], [16]; however, no quantitative experimental information is available.

Another phenomenon which has been found to influence the characteristics of the distribution of RBCs is that of RBC aggregation. RBCs have a propensity to reversibly bond to one another via interactions with proteins in the plasma under low shear forces. As the shear increases, the RBCs disaggregate and flow more independently. RBC aggregation is a complex phenomenon, and its effects on haemodynamics are still not completely understood. Contradictory studies on the influence of aggregation on apparent viscosity report an increase [23]–[26], a decrease [27], [28] or no effect [29], [30]. Enhanced aggregation is associated with a wide range of pathologies [31], but also only occurs in athletic species [32]. It is thus still unclear under what circumstances RBC aggregation is a deleterious or beneficial phenomenon [31]. Aggregation has been observed to increase radial migration of RBCs, enhancing CDL widths [6], [28], [33] and altering distributions of cells downstream in bifurcations [12]. RBC aggregation has also been reported to increase plasma skimming in individual bifurcations [12], [34], [35], but its effect in serial bifurcations has not been reported.

Despite the considerable amount of research into microhaemodynamics, the majority of studies report on the flow in straight channels. Those studies which consider bifurcations tend to do so predominantly in terms of bulk parameters, and thus local differences within individual vessels are overlooked. Blood viscosity is highly dependent on haematocrit [36], hence given that local haematocrit concentrations can vary from zero to significantly over the mean haematocrit in a given vessel, the blood viscosity at a given location may differ significantly from that predicted from bulk parameters.

In order to understand the characteristics of the microvasculature of a given organ, numerical modelling is often used, as the large number of vessels and inherent multiscale characteristics make experimental measurement extremely challenging and limited in terms of accuracy. Significant advances in numerical approaches to modelling microvascular networks include the ability to estimate boundary conditions [37] and the inclusion of vessel wall dynamics [38]. In such models, plasma skimming and apparent viscosity are estimated based on the considerable *in vivo* characterisation of Pries [22], [39], [40] using empirical models. In order to develop microvascular network models further, so as to include local effects, detailed information on the interactions between RBC concentration and velocities must be included.

The present study represents the early stages in the development of an experimental approach to better characterise local haemodynamics on the microscale. High resolution local concentration and velocity distributions of RBCs flowing in a sequentially bifurcating microchannel are reported under a number of flow conditions and the influence of RBC aggregation is analysed. The results highlight the considerable complexity in microvascular networks and the importance of accounting for local effects.

## Methods

### Experimental methods

#### Preparation of erythrocyte samples

The study was approved by the South East London Research Ethics Committee (Reference:10/H0804/21) and informed written consent was obtained from volunteers. Human blood was collected via venepuncture and mixed with 1.8 *mg/ml* EDTA to prevent coagulation. The samples were separated via centrifugation and the plasma and buffy coat were aspirated. The RBCs were then washed twice in phosphate buffered saline (PBS) and resuspended at the desired haematocrit. Dextran 2000 at 5 *mg/ml* was added to induce RBC aggregation for the Dextran samples. All samples were used within 4 hours of extraction, or were refrigerated and used within 3 hours after returning to room temperature.

#### Microfluidic channel fabrication

The microfluidic channels comprised a long straight section (∼10 *mm*) followed by two sequential 90° bifurcations, separated by 5 channel widths (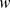). The cross sectional area of all branches was 50×50 *µ* *m*, giving an aspect ratio of unity.

The microfluidic devices were fabricated by bonding polydimethylsiloxane (PDMS) channel structures to glass microscope slides. Channel designs were produced in AutoCAD (Autodesk, USA). Standard photolithography techniques were used to produce a silicon (Si) master from which PDMS channel structures were cast. The process is summarised in the following: a chrome mask (JD Photo Tools, UK) was used to pattern a 1.8 *µ* *m* thick layer of positive tone photoresist (Microposit S1818: Rohm and Hass, USA) spun onto a 100 *mm* Si wafer (Compart Technology Ltd, UK). The exposed photoresist was developed using Microposit MF319 (USA) and hardbaked on a hotplate at 115*°C* for 5 *mins*. The wafer was then mounted on a ceramic carrier wafer and dry etched using an STS-DRIE system running the Bosch process; with the patterned photoresist acting as a protective etch mask. Following etching the photoresist was removed in acetone leaving the patterned wafer. The Si wafer was passivated by immersing in a 1% solution of tridecafluoro(1,1,2,2 tetrahydrooctyl) trichlorosilane in toluene; this allows subsequent release of cured PDMS from the Si master. A 10∶1 mixture of degassed PDMS was poured onto the wafer and baked overnight at 65*°C* until fully cured. Individual PDMS devices were then cut from the cured PDMS block using a razor blade and inlet and outlet holes punched using a biopsy punch (Technical Innovations, USA). The PDMS structures and the glass slides were both rinsed in IPA and blown dry prior to plasma treatment using a BD-20 Laboratory Corona Treater (Electro-Technic Products Inc., USA). Glass and PDMS were then brought into contact within a few seconds of treatment and left in an oven overnight at 65°C to fully bond.

#### Experimental setup

**Figure 1:**
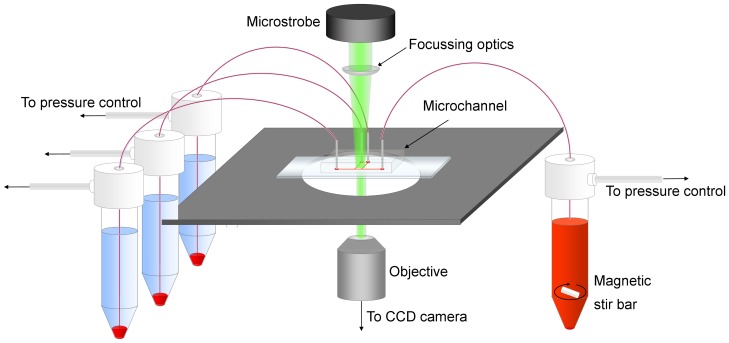
Schematic of the experimental setup. The blood sample is stirred continuously and perfused via pressure control. Focussed microstrobe illumination is used to acquire images of the flow with a CCD camera.

Figure 1 shows a schematic of the experimental setup. The sample was placed in a 15 *ml* centrifuge tube and mixed with a magnetic stir bar to allay the effects of sedimentation and aggregation in the feed reservoir. All reservoirs were connected via polyethylene tubing to the microchannel and the pressure in each reservoir was regulated using a multi-channel pressure regulator (MFCS-8C: Fluigent, France), which allowed accurate control of the applied pressure between 0 *mBar* and 1300 *mBar*. The microchannel was placed on an inverted microscope (DMILM: Leica, Germany) with a 10× air objective (*NA* = 0.25), focussed on the centre of the channel and illuminated with a green LED microstrobe. An *XYZ* micrometre stage and plano-convex lenses were used to focus the illumination on the region of study. A Hamamatsu C8484-05C CCD camera (Hamamatsu, Japan) was used to capture image pairs at 3 *Hz*, with the time between frames in each pair varied between 1, 2, 4 and 8 *ms* depending on the flow rate. The camera and pump were both controlled via PC and in-house written LabVIEW (National Instruments, USA) code.

For each case, the blood was perfused at high flow rate with the stir bar on, in order to ensure RBCs were distributed throughout the channel and connecting tubes. The flow rate was then decreased over a period of 5 *s* and the stir bar turned off. Image acquisition was triggered 20 *s* after flow reduction for a period of 20 *s*, after which the flow rate was returned to a high value and the stir bar was turned back on. All experiments were carried out at room temperature.

### Haematocrit distribution

All post processing was carried out using Matlab (Mathworks, USA) and compound uncertainties were estimated using the chain rule method of Kline and McClintock [41].

**Figure 2:**
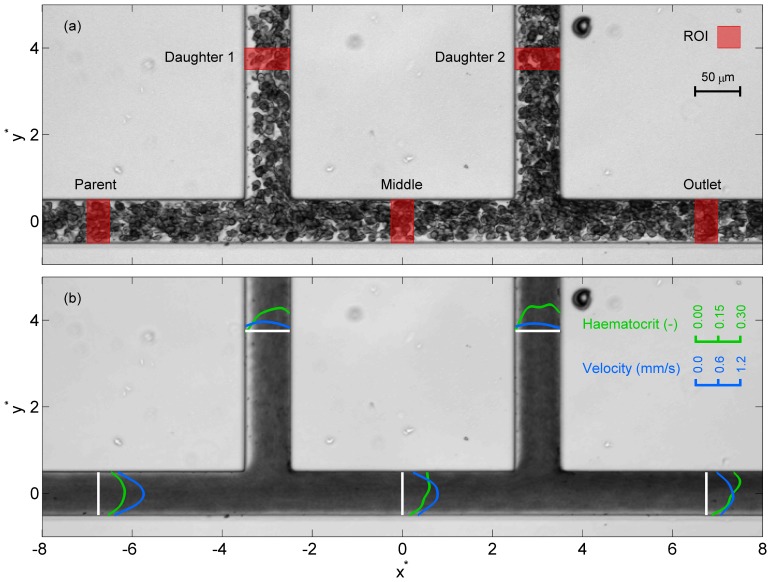
Sample image of the sequential bifurcation. (a) Sample instantaneous image of aggregating blood sample with regions of interest (ROI) marked. Blood enters through the parent branch (left of figure) and exits through all other branches. (b) Time averaged image over a period of 20 seconds. Superimposed profiles show haematocrit (green) and velocity (blue) profiles in the ROI. The co-ordinate system is defined such that (0,0) is in the centre of the parent branch equidistant between the two daughter branches.

For each case, a time-averaged haematocrit distribution was calculated using a refinement of a technique described previously [12]. The approach is based on the fact that the amount of light transmitted through the blood will decrease as the haematocrit increases. Thus, a mean image (in which each pixel value is the mean at that location of 120 images - using both images in each image pair), is calculated for each case to give a time-averaged distribution. The acquired images were first pre-processed to correct for illumination and orientation differences between cases (see Information S1 for details). Figure 2 shows sample instantaneous and mean images for a Dextran case. The co-ordinates 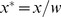 and 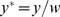 describe the channel dimensions in terms of the width 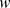. The intensity of each pixel in the mean image (Figure 2b) must then be related to the haematocrit at that location.

Conversion of pixel value to haematocrit was carried out by perfusing blood samples at haematocrits of 0.05–0.40 and acquiring images in a long straight section of the microchannel. For each haematocrit, Dextran and PBS blood samples at low, medium and high flow rates were considered. The average intensities were compared to the feed haematocrit and fitted to an exponential curve. Full details of the processing stages can be found in Information S2.

Haematocrit profiles for the branches in the *xy* plane were calculated as the mean over the regions of interest (ROI) indicated in Figure 2a. The average haematocrit in each branch was calculated by integrating the haematocrit profile. The average parent branch haematocrit was 0.158±0.009 for the Dextran data and 0.171±0.004 for the PBS data (weighted mean ± standard deviation).

### Velocity

The same images of the RBCs flowing in the microchannel were used for calculating the velocity in the channel using micro-particle image velocimetry (µPIV) algorithms. Each branch was selected separately and processed using multi-pass ensemble PIV using the freeware package JPIV (www.jpiv.vennemann-online.de). Three passes with interrogation windows (IW) of height 8 pixels and width of 64, 16, and then 8 pixels were used. This gave a final IW size of 4.8 µm, with a 50% overlap between interrogation windows, yielding a vector spacing of 2.4 µm. Invalid vectors were identified using the normalised median test [42] and were replaced by the median of the surrounding vectors. The average number of invalid vectors was 0.003% and 0.064% for the Dextran and PBS data respectively.

In µPIV processing, the finite depth of field results in an underestimation of the velocity profile, as tracers/RBCs away from the central plane contribute to the correlation peak. Poelma et al. [43] investigated the effects of channel width and microscope objective on velocity underestimation in PIV analysis of blood flow. An *in silico* model in their study predicted that the ratio of the measured velocity to true velocity at the centreline, 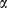, tended towards ∼0.66 for lower magnification and for smaller channels. In the present study, the channel width of 50 µm and 10× objective (*NA* = 0.25) suggest that a factor of 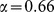
is appropriate. Hence, the velocity vectors were divided by this factor to adjust for out-of-plane effects.

For the calculation of flow rate, velocity profiles were required which described the flow across the entire channel. Velocity profiles were calculated by axially averaging all of the velocities in the ROI. Uncertainty at each radial location was defined according to the standard deviation of the vectors within the ROI. As a result of diffraction at the channel wall, the first usable vector was 8 pixels (∼5 µm) from the wall. Due to their finite size, the velocity of RBCs will not necessarily tend towards zero at the wall, as cells that come in contact with the wall tend to roll along it. Given this phenomenon, a linear extrapolation of the three vectors closest to the wall was used to estimate a slip velocity. Although a linear extrapolation is likely an oversimplification, it was considered to be a more accurate assumption than applying the no slip condition. The profiles with the extrapolated wall values were fitted with a smoothing spline that was used to interpolate the velocity data onto the same resolution as the haematocrit data, i.e. 84 pixels across the channel width. The uncertainty in the profile was similarly linearly extrapolated to the wall and interpolated.

### Quasi-3D assumptions and flux calculations

The parent branch follows a long straight channel section of 200 channel widths; hence the results in this branch represent the equilibrium condition between radial migration and margination forces. As the channel has an aspect ratio of unity, it can be assumed that, under the assumption of negligible RBC sedimentation, the distributions of velocity and haematocrit will be the same in horizontal and vertical directions at equilibrium. Based on this assumption, *yz* plane distributions of haematocrit and velocity can be estimated in each of the ROI (in the daughter branches, it would in fact be the *xz* plane. However, for the sake of clarity, the following discussion refers only to the *yz* plane). The details of this analysis can be found in Information S3.

Based on the *yz* plane distributions of velocity and haematocrit, the volumetric flow rate and mass flow rate can be calculated for each branch, as described in Information S4. As a measure of the accuracy of the data, analysis of both mass and volumetric conservation can then be applied at each of the bifurcations separately (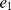, 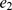) or for the whole channel (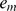), with 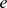 defined as the difference between the volumetric or mass flow rate in and out of the control volume (shown in Figure 2) as a percentage of the inflow. Table 1 lists the calculated errors. For the volumetric conservation (based only on the PIV) the errors are very small, with a maximum of 3.5% for 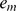 in the PBS case. The mass flux calculations do not overall have increased error, although a maximum of 5.9% is observed in the second bifurcation for the PBS case. These low deviations from continuity provide confidence that the proposed assumptions are appropriate for the present data.

**Table 1:** Errors in RBC mass continuity and volumetric continuity, shown as weighted mean and standard deviation, with weights defined from the reciprocal of the variance for each data set, calculated from the cumulative errors.

	Mass Continuity						Volumetric Continuity					
	*e*_1_ (%)		*e*_2_ (%)		*e*_m_ (%)		*e*_1_ (%)		*e*_2_ (%)		*e*_m_ (%)	
	Mean	SD	Mean	SD	Mean	SD	Mean	SD	Mean	SD	Mean	SD
**Dextran**	2.0	2.0	−5.9	2.5	−2.4	2.0	1.5	0.6	1.3	0.6	2.4	0.7
**PBS**	0.5	2.0	−3.2	1.8	−2.0	2.6	2.0	1.4	2.2	1.3	3.5	1.8

### Flux-flow curves

In the results section, the flux-flow curves calculated from the present data set are presented. The deviations from volumetric and mass continuity given in Table 1 lead to additional uncertainty in the fitting. Hence, the flux and flow data are first corrected, by enforcing conservation of mass in a manner similar to that carried out by Pries et al. [22]. The corrected flow ratio 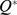 in, for example, the first daughter branch is given by
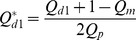
(1)


Where 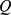 is the flow rate and the subscripts 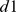, 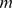 and 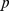 refer to daughter branch 1, the middle branch and the parent branch respectively. Similarly, for the middle branch
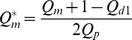
(2)


The same corrections were carried out in the second bifurcation and for the flux ratio, 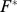, and uncertainties were quantified using the chain rule of differentiation [41]. The corrected data was fitted to the commonly used *logit* function described by Pries et al. [22]:
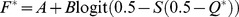
(3)


The term 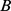 defines the sigmoidal shape of the curve and 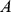 is an asymmetry parameter, indicating the difference between two branches of a bifurcation. 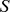 is related to the critical flow ratio, 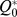, below which no RBCs will enter a given daughter branch according to 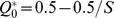.

In an asymmetric bifurcation, one branch takes the shape of the curve with 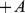, whilst the other branch takes the shape of the curve with 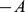. In order to efficiently calculate the fit, the flux and flow ratios in one branch are inverted, so that all data acts as if it were in one branch of the bifurcation. Non-linear regression is then used to fit the data to Equation 3, and the results for 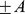 are given along with 95% confidence intervals for each parameter. The *R* ^2^ values for the fits were 0.98 or higher for all cases.

### Profile parameterisation

#### Velocity bluntness

In order to analyse the effect that flow rate has on the shape of the velocity profile, a bluntness index, 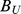 is defined, similarly to Alonso et al. [44], but modified for a square channel.

The analytical solution for the velocity, 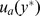, of a Newtonian fluid in the central plane of a long straight section of a square microchannel of width *w* is given by Bruus [45]:
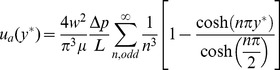
(4)where 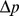 is the pressure drop along length L and 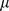 is the dynamic viscosity. 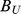 is then calculated for the experimental velocity profile 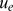 according to



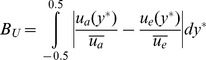
(5)If the measured velocity profile matched the analytical solution, 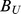 would equal zero. A flat velocity profile (plug flow) would yield 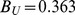. Thus 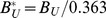, which yields 0 for a Newtonian fluid and 1 for plug flow. As 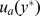 is exact, the uncertainty 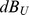 is given by
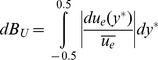
(6)


#### Haematocrit bluntness

There is no analytical solution available for the shape of the haematocrit distribution; hence shape parameters for the haematocrit are defined based on the distribution of haematocrit in the channel. The bluntness index 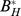 is defined based on the proportion of flow in the central third of the channel (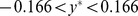). 
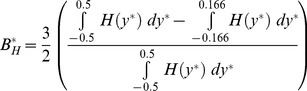
(7)


The uncertainty in the bluntness index, 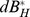, is calculated using the chain rule of differentiation. The 3/2 term yields a parameter which varies between 0 when all of the RBCs reside in the central third of the channel, and 1 when there is a uniform distribution of RBCs (a constant haematocrit distribution).

#### Haematocrit skewness

In the branches of the bifurcation other than the parent branch, the haematocrit profile 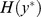 becomes skewed, as can be observed in Figure 2b. A haematocrit skewness index, 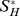, is defined according to
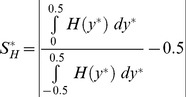
(8)in the middle and outlet branches, with the appropriate change of co-ordinate system in the daughter branches. The fraction gives the proportion of flow lying on the left hand side of a given branch along the direction of flow, with 0.5 subtracted so that 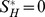 is a symmetric profile. The uncertainty 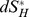 is calculated using the chain rule of differentiation.

## Results

### Parent branch

**Figure 3:**
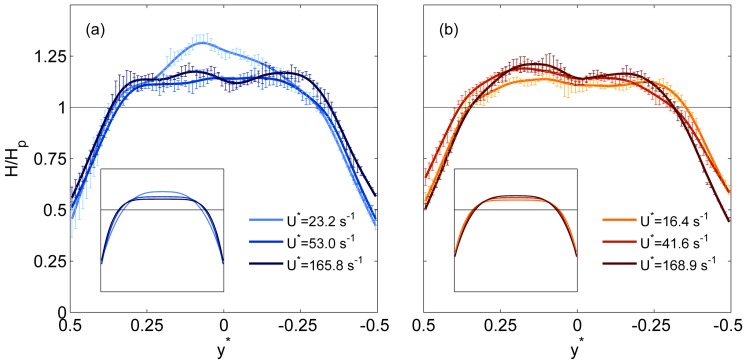
Parent branch haematocrit profiles. Sample haematocrit profiles in the parent branch for a range of 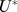. Lines show spline fits through the data and error bars show standard deviations. (a) Dextran data (b) PBS data. Uniform haematocrit distribution is indicated by the solid line. Insets show fits to Equation S14: axes on the insets are equal to the main figure.

As discussed previously, the parent branch represents the equilibrium condition, and the results should be comparable to previous studies in long straight glass capillaries or vessel sections. Sample haematocrit profiles are shown in Figure 3 for a range of normalised velocities 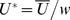 (equivalent to channel widths per second). For the Dextran cases (Figure 3a), it can be seen that at 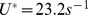, the haematocrit concentration in the channel centre was around 30% greater than the mean haematocrit in the branch. As 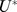 increased to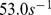, the distribution became more uniform, with a lower RBC concentration in the channel centre. For 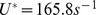 the profile was similar to that observed for 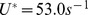. For all flow rates, the normalised haematocrit at the wall was approximately 0.5. It should be noted that this is a time-averaged distribution, and the instantaneous values varied between 0 and some greater value, as can be understood by considering the parent branch in the instantaneous image shown in Figure 2a. The inset in Figure 3a shows the haematocrit profiles fitted to Equation S14 in Information S4, which shows the trend of increasing bluntness more clearly.

In Figure 3b, equivalent profiles are shown for the PBS data, in which RBC aggregation is absent. For this data, the profiles show the inverse trend; the bluntness of the profile decreased as the flow rate increased. The three profiles appear to be fairly similar, however it can be seen that for 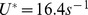, the normalised haematocrit in the channel centre is lower than for higher 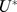. The inset shows this trend more clearly.

**Figure 4:**
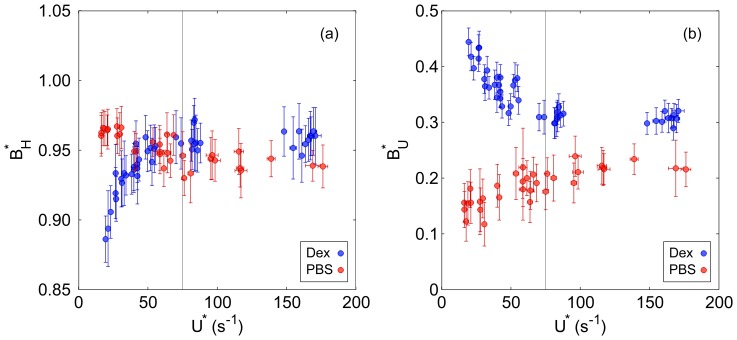
Haematocrit and velocity profile bluntness. (a) Haematocrit bluntness index, 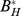 in parent branch against 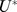. (b) Velocity bluntness index, 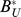 in parent branch against 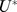. Error bars show one standard deviation.

These observed trends can be further analysed by considering the haematocrit bluntness index 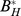 as a function of 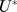, as shown in Figure 4a. It can be seen that as 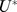 increases to approximately 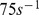, the Dextran data shows a rapid increase in bluntness, while the PBS data shows a steady decrease over the same range. For 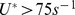, 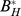 changes relatively little for both the Dextran and PBS data, but the former is blunter. The significance of the observed trends can be supported by the Spearman's rank correlation coefficient, *r*, and corresponding *p*-value. The rank correlation is selected due to the non-linear nature of the relationships observed in Figure 4. The correlation coefficients were 0.844 and −0.812 (*p*<10^−6^) for the Dextran and PBS cases respectively, highlighting the opposing directions of the trends.

To investigate the impact of the shape of the parent branch haematocrit profile on velocity, the velocity bluntness index, 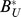, is shown in Figure 4b. Both Dextran and PBS data display the opposite trends observed for 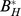 with 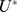: the velocity bluntness decreases with increasing flow rate for the Dextran case (*r* = −0.841, *p*<10^−6^), and vice versa for the PBS case (*r* = 0.819, *p*<10^−6^). Comparing 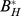 directly to 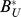 confirms the inverse relationship between the indices for both Dextran (*r* = −0.827, *p*<10^−6^) and PBS (*r* = −0.746, *p*<10^−6^) data.

It should be noted, however, that 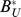 is significantly greater for the Dextran data, even when 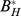 is also greater, suggesting an influence of RBC aggregation even at high flow rates. To demonstrate this, correlations were calculated for 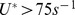, indicated by the grey line in Figure 4. Neither 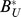 or 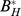
was found to vary significantly with 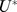 (*p*>0.1). One can thus compare the mean values of the Dextran and PBS cases using the Wilcoxon rank sum test. This confirmed that both velocity and haematocrit bluntness were significantly greater for the Dextran data (*p*<10^−5^).

### Daughter branches

The regularly branching geometry of the vasculature introduces complexities in local haematocrit distributions, and thus it is necessary to consider what happens around and downstream of bifurcations. As can be seen in Figure 2b, the haematocrit distribution in all branches other than the parent branch is skewed, i.e. the distribution of RBCs is not symmetric about the channel centre line. Additionally, the velocity profiles can be observed to be skewed in the opposite direction. In order to investigate the effect of the flow ratio on the shape of the profiles, and their propagation through the channel, four Dextran cases were selected in which the flow ratio 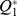 varied, but 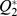 and 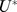 were similar. Table 2 lists the parameters for the four cases.

**Table 2:** Flow parameters for the selected cases analysed in Figures 5 and 6.

Case	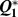	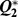	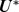 (s^−1^)
*i*	0.13	0.35	17.1
*ii*	0.20	0.37	23.9
*iii*	0.32	0.44	26.5
*iv*	0.39	0.43	26.2

**Figure 5:**
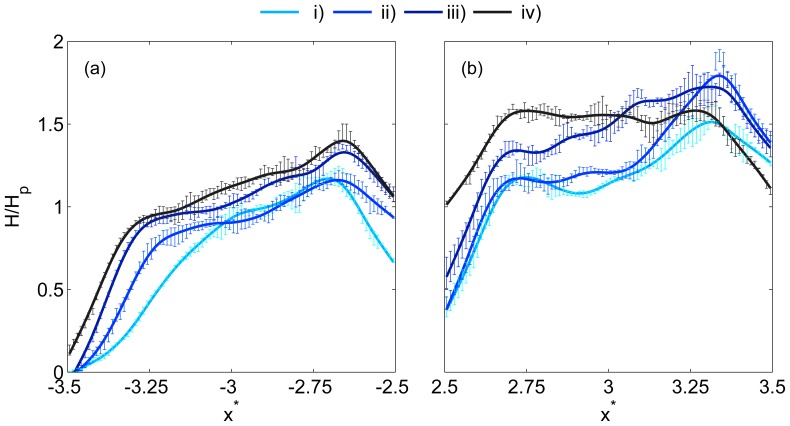
Daughter branch haematocrit profiles. Sample haematocrit profiles in (a) daughter branch 1 and (b) daughter branch 2. See Table 2 for details of flow ratios. Profiles are indicated with a smoothing spline for clarity and error bars show one standard deviation.

Figure 5 shows haematocrit profiles in daughter branches 1 and 2. For cases *i–iii* in daughter branch 1 (Figure 5a), it can be seen that there is a small true ‘cell-free layer’ at the channel wall closest to the feed (parent) branch, 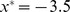, but for case *iv*, the haematocrit at the wall is non-zero. The slope of the haematocrit profile away from the 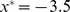 wall increases as the flow ratio increases. On the opposite wall of daughter branch 1 (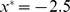), the trend in the slopes of the profiles is reversed and the haematocrit at the wall is very high, with a minimum of 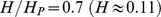 for case *i*, increasing to 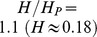 for case *iv*. In daughter branch 2, (Figure 5b), the flow ratios (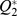) are similar for all cases, but the profiles differ significantly, particularly when compared to case *iv* in daughter branch 1 (which has the same flow ratio). Case *iv* in daughter branch 2 has an almost symmetric profile, which is slightly skewed towards the wall closest to the feed (middle) branch,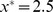, with high wall haematocrit on both sides of the channel. For case *iii*, in which the flow ratio in the preceding bifurcation is lower, the profile is skewed towards the opposite wall (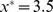). Cases *i* and *ii* both exhibit a dip in the haematocrit in the channel centre, but are otherwise comparable to case *iii*.

**Figure 6:**
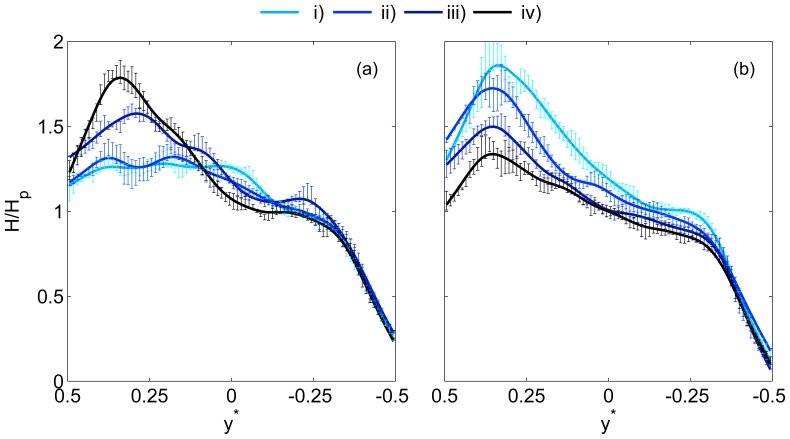
Middle and outlet branch haematocrit profiles. (a) Sample haematocrit profiles in the middle branch and (b) in the outlet branch. See Table 2 for details of flow ratios. Profiles are indicated with a smoothing spline for clarity and error bars show one standard deviation.

The causes of these profile shapes can be explained in part by analysing the haematocrit profiles in the middle branch (Figure 6a). In this branch, RBC concentration is greatest near the wall adjacent to the daughter branches (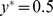). This is a result of the geometry of the bifurcation and the increased haematocrit in the channel centre in the parent branch, as streamlines in the channel centre are drawn towards the apex of the bifurcation. This profile persists along the middle branch such that the streamlines closest to daughter branch 2 have a high haematocrit 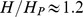, as opposed to the low haematocrit in the equivalent location (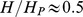) in the feed vessel for the first bifurcation (parent branch, see Figure 3a). For the higher first bifurcation flow ratios (cases *iii* and *iv*), more of the central streamlines are drawn towards the apex and hence the haematocrit close to 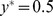 is greater. This then translates to an increased haematocrit at 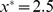 in daughter branch 2 and the corresponding profile shapes observed in Figure 5b.

Figure 6b shows the haematocrit profiles in the outlet branch. Interestingly, despite the similar second bifurcation flow ratios (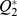), the profiles vary more than in the middle branch. For case *iv*, the wall haematocrit at 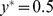 is 1, and this increases for lower 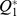 values (and 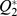 values). This trend is visible in the profiles in the channel centre as well, wherein the greatest haematocrit is observed for case *i*. It should be noted that the profiles have similar shapes, but are scaled due to plasma skimming in the sequential bifurcation reducing the haematocrit in the outlet branch.

**Figure 7:**
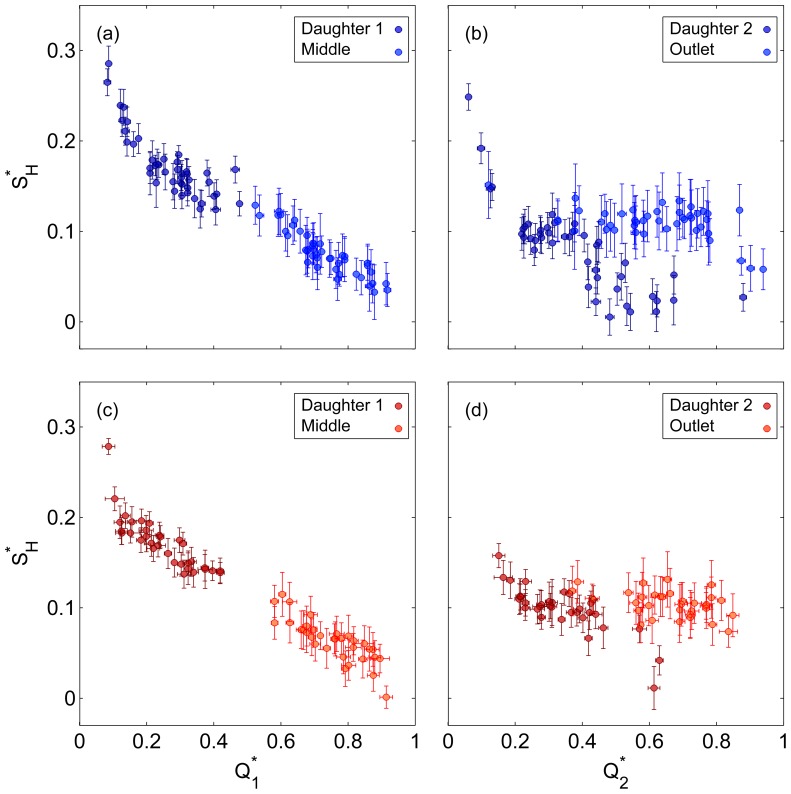
Skewness of the haematocrit profiles. Haematocrit skewness index, 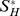, as a function of flow ratio in (a) Bifurcation 1 (b) Bifurcation 2. Error bars show one standard deviation.

In order to consider broader relationships between the flow ratio and the shape of the haematocrit profile, the haematocrit skewness index 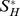 is considered in Figure 7 for both bifurcations. It should be noted that the definition of 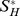 uses an absolute value so that the data in separate branches can be considered together; if the magnitude was omitted from Equation 8, then 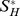 would be negative in both daughter branches and positive in the middle and outlet branches.

Despite the difference in geometric configuration between the first daughter and middle branches, 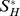 in the first bifurcation collapses very strongly onto a single curve. For 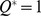, 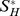 should tend towards zero, which is confirmed in Figures 7a and c. As 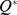 approaches zero, the proportion of RBCs on one side of the channel should approach one, yielding 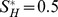. The relationship between 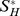 and 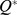 seems to be linear as 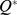 decreases from 1 to 0.2, then 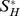 increases rapidly for lower flow ratios towards the limiting value of 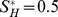. For both Dextran and PBS cases, the observed trends are highly significant (*p*<10^−6^). The strong similarity between both branches supports the idea that, at least for a single bifurcation, the geometry is not a key parameter [16].

However, in the second bifurcation (Figure 7b,d), the difference between the second daughter and outlet branches is notable. In the latter, the skewness index remains around 0.1. No correlation is observed between the skewness and flow ratio for the Dextran case (*p* = 0.21) and only a small correlation is observed for the PBS case (*p* = 0.024). This contrasts with the first bifurcation in which the skewness varied very significantly with flow ratio. In the second daughter branch, the low flow ratio limit appears to be the same as for the first bifurcation, for 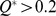, the skewness appears to decrease towards 0 as in the first bifurcation. Significant relationships are observed for both Dextran and PBS cases (*p*<10^−5^). However, the data is rather scattered compared to the first bifurcation, implying an effect of the flow ratio in the first bifurcation on the shapes of the profiles in the second bifurcation, as observed in Figures 5 and 6.

**Figure 8:**
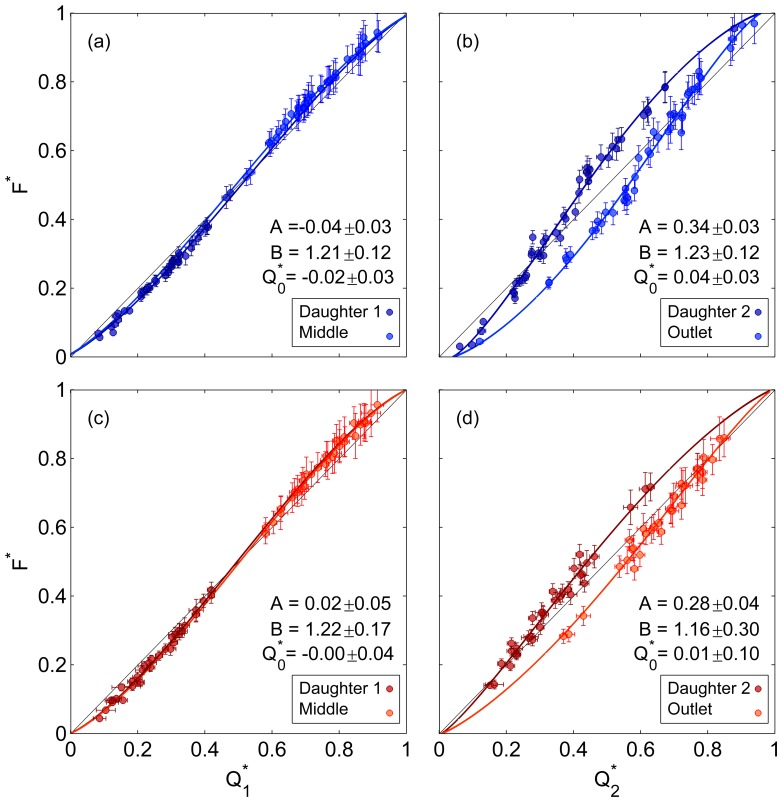
Flux-flow curves. Flux-flow curves with empirical fits (a) Bifurcation 1, Dextran (b) Bifurcation 2, Dextran (c) Bifurcation 1, PBS (d) Bifurcation 2, PBS. Error bars show one standard deviation. Parameter values and 95% confidence intervals are given for each fit.

By integrating the quasi 3D haematocrit and velocity profiles, plasma skimming in the serial bifurcations can be investigated. Figure 8 shows the flux-flow curves for the sequential bifurcations with sigmoidal fits as described in the methods section. The 95% confidence intervals on the parameters give an indication of significance when comparing different cases. Deviations from the 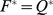 line (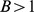) imply the presence of plasma skimming and can be seen in both bifurcations for Dextran and PBS data. In the first bifurcation, the Dextran (Figure 8a) and PBS (Figure 8c) data show very similar curves, as verified by the similar fitting parameters for 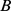. The critical flow ratio,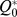, below which no cells would enter the daughter branch, was not found to be significantly different from zero for both Dextran and PBS data in the first bifurcation. Furthermore, the asymmetry parameter *A* was very small, indicating that daughter branch 1 and the middle branch have similar characteristics. However, in the second bifurcation (Figures 8b and d) there is a strong asymmetry between daughter branch 2 and the outlet branch for both the Dextran (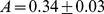) and PBS (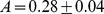) data. The critical flow ratio for the Dextran case in the second bifurcation was 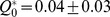, which is significantly greater than zero, but was 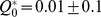 for the PBS data. Despite the relatively large confidence intervals on the fitted parameters, it is clear that the second bifurcation has significantly different characteristics to the first, as concluded from direct analysis of the haematocrit profile shapes. RBC aggregation also seems to slightly enhance this difference.

## Discussion

The present study provides high resolution quantitative data on RBC concentrations, yielding further insight into the interactions between local haematocrit distributions, the geometry and the flow conditions.

The results in the parent branch showed that for both data sets (Dextran and PBS), the bluntness of the haematocrit and velocity profiles were very strongly negatively correlated, as reported by Goldsmith [46]. For aggregating blood (Dextran data), at low flow rates, the RBC concentration was very high in the channel centre, due to enhanced radial migration and possibly synaeresis. As a result, the viscosity in the channel centre was greater than at the walls, and the local velocity was reduced relative to that of a uniform RBC distribution. As the flow rate increased, RBC disaggregation and margination led to a blunter haematocrit profile and correspondingly a less blunt velocity profile. For the RBCs suspended in PBS, increasing flow rate resulted in a less blunt haematocrit distribution, as radial migration increases with shear rate near the walls [47]. The trends observed in Figure 4 show the relative importance of the effects of shear-induced radial migration and RBC aggregation. The former seems to moderately affect the RBC distribution in a linear fashion, as evidenced by the continual decrease in haematocrit bluntness with increasing flow rate for the PBS data. RBC aggregation has a much stronger effect at low flow rates, but the bluntness of the Dextran case changed relatively little for 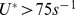. However, the bluntness of the haematocrit profile was significantly increased by the presence of aggregation at high flow rates. It should be noted that 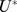 corresponds to the ‘pseudo-shear rate’ used by other researchers. The present data shows that RBC aggregation has a significant effect for 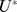 up to 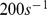, further extending the previous range reported *in vivo;* 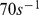 in venules [48] or 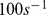 in arterioles [4]. For comparison with shear rates reported in constant shear viscometers, note that the average shear under Poiseuille flow is 16/3 times greater than the pseudoshear and increases as the profile is blunted. Despite the trend observed between haematocrit and velocity bluntness, the latter was higher for the Dextran data than for the PBS data, even when the haematocrit bluntness was also higher. In the presence of RBC aggregation, even at high flow rates, the interconnections between cells limit their independence, resulting in ‘plug-like’ flow in the channel centre.

The phenomenon of plasma skimming in single bifurcations has been investigated in depth using bulk measurements of RBC concentration and flow rate. The diameter of the vessels/channel has been identified as a critical parameter, with plasma skimming reported to increase significantly as the conduit decreases in size [16], [22], although diameter has also been suggested to be of little significance [13]. The present data shows rather more plasma skimming than reported by Fenton et al. [16] for 50 µm bifurcations. However, their flow rates were higher and their data more scattered. Given the range of parent branch flow rates, the fact that the data fit so well to the *logit* function line supports the conclusion that parent branch flow rate is not a key parameter in plasma skimming, particularly when compared to the flow ratio. In the second bifurcation, there is considerable asymmetry between daughter branch 2 and the outlet branch. For flow ratios over 0.3, disproportionately more RBCs enter the daughter branch in both Dextran and PBS data. The reason for this is clearly shown in Figure 6a, as skewing of the haematocrit profile in the middle branch results in a high haematocrit concentration on the side of the separation surface which feeds the second daughter branch. As with the first bifurcation, the data collapses well onto the *logit* fit, despite the range of absolute flow rates and flow ratios in the first daughter branch. It should be noted that 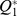 values over 0.5 were not considered for the present data set. However, for any flow ratio in the first bifurcation, the haematocrit profile in the middle branch would always be skewed in the same direction due to the local geometry. Indeed, the haematocrit skewness index shown in Figure 7a increases as the proportion of flow entering the middle branch decreases, implying that the asymmetry seen in Figure 8b and d would persist for all 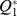. Figure 7b further shows that the skewness in the second daughter branch is reduced relative to the first daughter branch, but that the skewness in the outlet branch is higher than for the middle branch for flow ratios greater than 0.5. These complex relationships between the branches highlight the importance of considering local characteristics of RBC concentration.

Despite having a significant influence on the characteristics of the flow in the parent branch, RBC aggregation was observed to have less impact on the asymmetry observed in the bifurcations. The haematocrit skewness index showed no discernible difference between the Dextran and PBS cases, and the extent of plasma skimming in the first bifurcation was also similar. In the second bifurcation, there was a clear increase in asymmetry and slightly more plasma skimming in the Dextran case, although the reduced range of 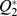 increased the confidence intervals for the PBS data. In a previous study in high aspect ratio (100×40 µm) straight T-bifurcations [12], we found negligible plasma skimming for PBS data (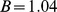), but moderate plasma skimming for the Dextran data (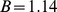); a level comparable to the present data. This difference may be due to the difference in geometry (aspect ratio, channel width, straight-T) and/or the lower flow rates used in that study, which would further enhance the impact of aggregation.

The haematocrit distribution technique employed in the present study is founded on the assumption that the time-average pixel intensity at a given location is related to the haematocrit in a predictable way. The calibration stage (Figure S1) showed that there is a certain amount of scatter about the best fit line, with an uncertainty of ∼0.014. However, the normalisation of the images largely removed the random differences. This correction should not significantly affect the shape of the haematocrit profiles. The use of empirical equations (S3 and S4) to convert the feed haematocrit to a channel haematocrit is a further assumption, which is likely to influence the absolute values of haematocrit reported here. Ideally, the output from each channel would have been collected and the haematocrit measured directly. However, in the presence of RBC aggregation, the required experimental protocol precluded such an approach. The flow rates used in the present study range from 0.15–1.28 *µ* *l/min*, which over a time period of 20 *s* equates to volume of 0.05–0.43 µ*l* split between the four outflow branches. Separating the sample for each branch under the current conditions was thus not feasible for the present study. Furthermore, these very low flow rates, combined with the short time scales required due to aggregation precluded measuring the flow rate, which would have been good way to validate the estimated velocities and evaluate the efficacy of the adjustment based on the data of Poelma et al. [43].

The depth of correlation (DOC) in PIV processing can be estimated according to the analytical equation of Olsen and Adrian [49]. For the present system, if standard laser illumination and 1 µm particles had been used, the DOC would be 38 µm. Although the equation is not directly applicable to RBC images, being derived on the basis of sparse Gaussian particles, the large dimensions of RBCs compared to tracer particles indicates a larger depth of field, which would encompass the entire channel depth. Additionally, Rossi et al. [50] reported that the true depth of correlation is often greater than the one estimated using the aforementioned equation [49]. Thus it is likely that the PIV velocities, intended to yield the velocity in the central plane, are significantly underestimated.

For the data presented in this paper, we adjusted the PIV velocity based on the model of Poelma et al. [43], using 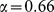. This value of 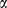 is the theoretical limit at which point all particles contribute equally to the estimated velocity for Poiseuille flow. Although this value was defined for round channels, the velocity profile in square channels is not significantly different. Indeed, the theoretical limit for a square channel based on the analytical solution [45] is 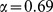, a difference of only 4% which is independent of flow rate. A more important difference may arise from the fact that this value could be expected to increase as the bluntness of the velocity profile increases. In order to investigate whether this would affect the conclusions drawn in the present study, the profiles fitted to Equation S17 were assessed to calculate 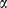 for each case. The adjusted normalised velocities, 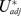, were then compared to 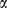. For the PBS data, 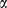 did not correlate with 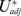 (*p* = 0.36) and had an average of 0.70±0.01. For the Dextran data, 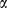 did correlate with 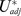 (*r* = −0.749, *p*<10^−6^) and decreased from 0.77 to 0.75 as 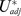 increased. The conclusions of the statistical analysis on the bluntness parameters were not altered by this process and all significant trends remained so. Furthermore, the assumption of symmetry in the *z*-axis would imply that the same value of 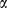 should be applied in all channels, and thus would not affect 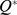 or 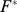. In the absence of the necessary data to rigorously validate this adjustment, the simpler single-value adjustment was preferred for the main analysis in the present study. However, for reference we provide in Figure S2: the bluntness parameters shown in Figure 4 plotted against 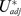. It can be seen that the shape of the observed trends is not significantly affected. Nonetheless, exact values of 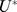 reported in this (and any blood PIV based studies) must be considered with caution if applied in numerical analyses.

The linear extrapolation of both haematocrit and velocity profiles to the wall is another assumption which may influence the results. One could argue that at the wall, the RBC concentration and velocity must both be zero. However, due to the finite size of RBCs such an analysis would be inaccurate. Considering Figure 2, it is clear that the wall haematocrit does not reach zero at all places. With regards to velocity, the RBCs which do flow close to the wall can be observed to roll along it in videos of flow. Furthermore, the shape of the velocity profile (not shown) indicated a non-zero wall velocity. Although the true wall velocity probably lies somewhere between zero and the linearly extrapolated value, the latter was chosen to be more suitable. Indeed, the fitting of Equation S17 under the assumption of zero wall velocity (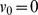) resulted in more than twice the average deviation from the fitted line.

The quasi-3D extension of the data required that sedimentation was negligible in the channel. Alonso et al. [44] analysed RBC sedimentation and found a negligible amount for data with the equivalent of 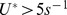 over a period of 5 minutes in a 59 *µ* *m* channel. Due to the significantly lower experimental time and higher flow rates used here, it seems reasonable to assume that sedimentation was not a factor over the period of data acquisition.

Despite the potential limitations, the low reported values of the error in volumetric and mass conservation reported in Table 1, combined with the minimal scatter observed in Figure 8, support that the haematocrit quantification carried out in the present work is sufficiently accurate.

The channel haematocrit (∼0.16) used in the present study is significantly lower than that which would be found in large arteries (∼0.45). However, due to the Fåhraeus effect, haematocrit is substantially reduced in smaller vessels. There is limited data on humans, but data from a cat mesentery [2] shows haematocrits generally in the range 0.1*–*0.2 for 50 µm diameter arterioles. Hence it is reasonable to call the haematocrit used in the present study ‘physiological’.

The present study has shown the significant variability of local characteristics exhibited by microscale blood flow. As an example, the large range of wall haematocrits shown in Figures 5 and 6, from approximately 0 to 1.4 times the average haematocrit in the parent branch, implies that estimates of wall shear stress (the product of viscosity and shear rate at the wall) which assume 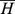 or 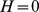 at the wall, as is common [51], could be very inaccurate. The corresponding skewed velocity profiles would further complicate this effect. In the parent branch, the varying bluntness in velocity profiles would also alter the wall shear rate relative to the mean flow.

A potential application of this work, or extensions thereof, is as a validation tool for computational methods which seek to reproduce microvascular blood flow. There is a large amount of research effort put into modelling discrete flow of RBCs [52]–[56], but experimental validation of these models is limited or lacking. Recent reviews have highlighted the need for increased communication between modellers and experimentalists [57], [58] and more detailed experimental data [59]. The present study provides an ideal initial data set for validation of these models. Additionally, microvascular network flow analyses rely on empirical models based on bulk parameters for haematocrit and viscosity [37]–[39]. Although wider coverage of the parameter space is required, the present approach could yield more detailed models, which may, for example, be used to analyse the effect of increased aggregation on the network behaviour. Although *in vitro* data uses idealised geometries and simplified dynamics, it allows accurate control of the parameter space and a resolution which is not currently possible *in vivo*.

## Conclusions

Understanding the fundamentals of haemodynamics is of vital importance to clinicians and researchers when analysing blood flow in physiology and pathology. However, many of the basic aspects of blood flow are commonly overlooked, such as the local distributions of RBCs and the effects of aggregation. The present study aimed to provide further insight into these important parameters using a microchannel representing a sequentially bifurcating arteriole. Quantitative parametric data on the shapes of the RBC distribution in complex geometries have been provided for the first time. Strong correlation between the haematocrit and velocity profiles were observed as a function of the flow rate in the parent branch, which provide further insight into the effect of RBC aggregation on the flow. In the daughter branches, strongly skewed haematocrit distributions were observed, which could have a significant influence on estimation of haemodynamic parameters such as apparent viscosity and wall shear stress. The two branches of the first bifurcation showed very similar characteristics with respect to flow ratio, but in the second bifurcation the asymmetry between the two branches was greatly increased. The causes for this were elucidated by considering individual haematocrit profiles.

This paper reports results for a specific subset of the vast parameter space which must be investigated in order to fully comprehend the complexities of microhaemodynamics. The fluid parameters which require further consideration are a range of haematocrits, different levels of RBC aggregation and flow rates relative to the geometry. Key geometrical considerations which should be considered include the distance between sequential bifurcations and analysis of a bifurcation on the opposite side to the first. The geometries should also be considered at a range of sizes, as the relative channel to RBC size is a key parameter. Compilation of such data could lead to the development of a parametric model of local haemodynamic effects which could be applied in network modelling studies.

## Supporting Information

Figure S1
**Haematocrit - intensity calibration.** Haematocrit- intensity calibration. Result of the calibration, showing haematocrit against normalised image intensity. Dots show 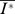 against 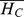, with error bars showing 1.96 standard deviations. Grey line shows best fit to Equation S7 based on non-linear regression. The black line shows fitted calibration curve after minimisation, 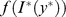 with parameters calculated as described in the text.(TIFF)

Figure S2
**Haematocrit and velocity profile bluntness against adjusted normalised velocity.** This figure is similar to Figure 4, but shows 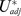 rather than 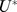. (a) Haematocrit bluntness index, (b) Velocity bluntness index. Error bars show one standard deviation.(TIFF)

Information S1 **Image pre-processing.** Details of the image processing methods applied prior to PIV and image intensity analysis.
(PDF)

Information S2 **Haematocrit calculations.** Description of the method applied for calibrating the haematocrit-intensity relationship and application of the technique to the bifurcations.
(PDF)

Information S3 **Quasi-3D assumptions.** Explanation of the mathematical formulation of the assumptions used to infer three-dimensional distributions of haematocrit and velocity.
(PDF)

Information S4 **Volumetric and RBC mass flow rate.** Description of the calculations of flow rate parameters from the acquired data and analysis of deviations from continuity.
(PDF)
