# Supplementary figures and images for: Spatial Distributions of Red Blood Cells Significantly Alter Local Haemodynamics
Source: PLoS One. 2014 Jun 20;9(6):e100473. doi: 10.1371/journal.pone.0100473 (PMC4065105; doi:10.1371/journal.pone.0100473)

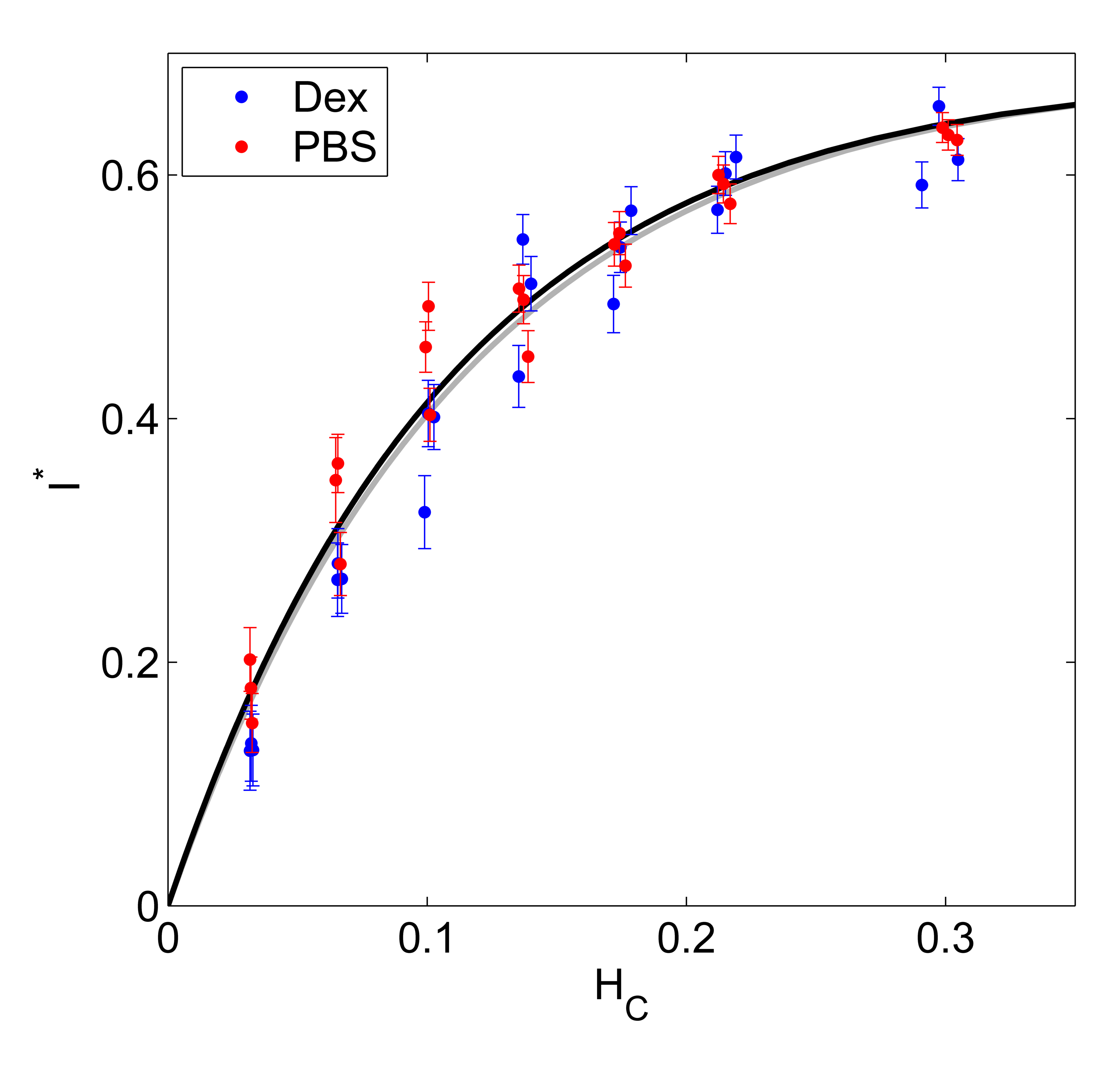

Supplement: Figure S1 — Haematocrit - intensity calibration. Haematocrit- intensity calibration. Result of the calibration, showing haematocrit against normalised image intensity. Dots show against , with error bars showing 1.96 standard deviations. Grey line shows best fit to Equation S7 based on non-linear regression. The black line shows fitted calibration curve after minimisation, with parameters calculated as described in the text. (TIFF) [file pone.0100473.s001.tiff]

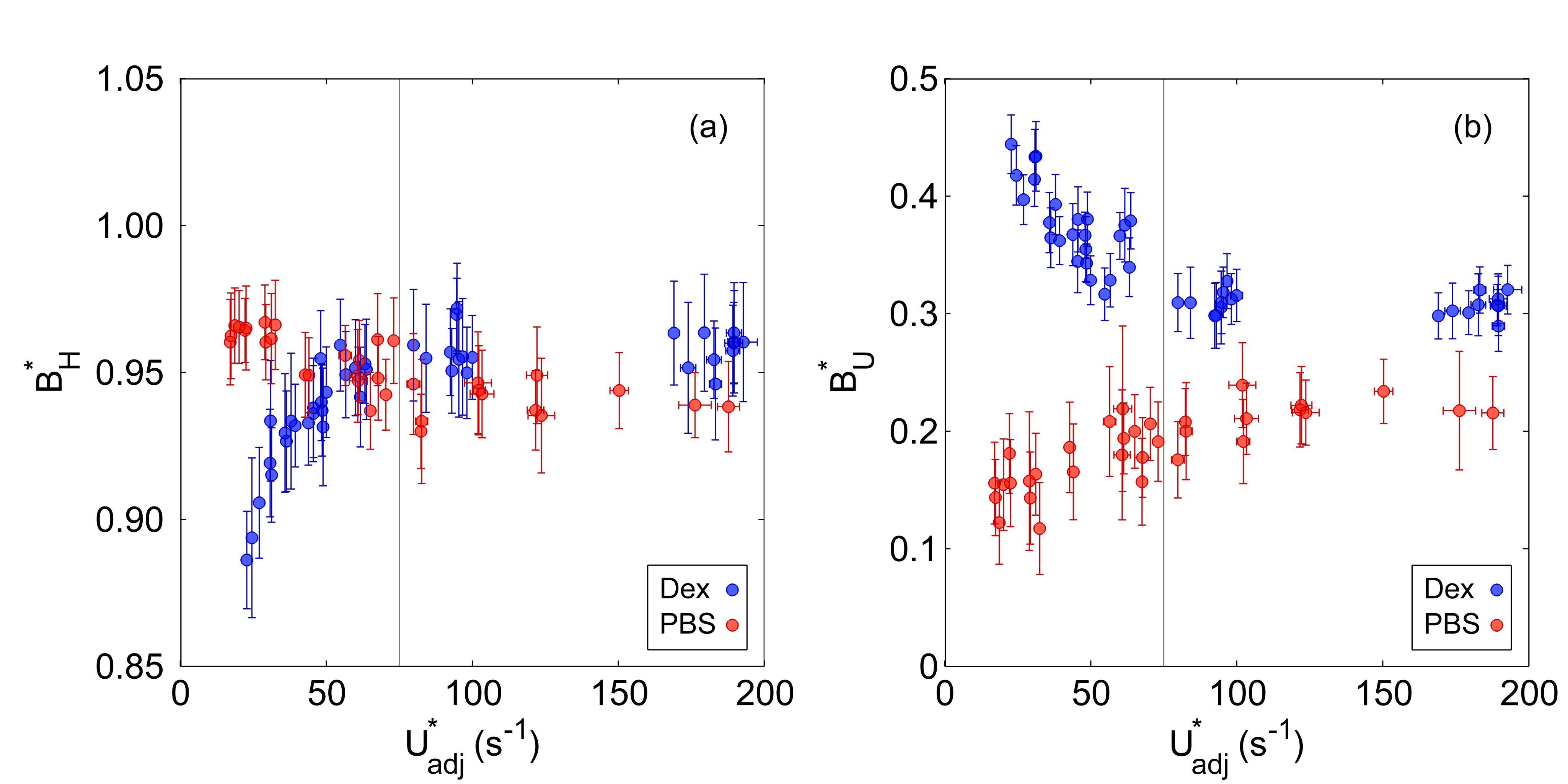

Supplement: Figure S2 — Haematocrit and velocity profile bluntness against adjusted normalised velocity. This figure is similar to Figure 4, but shows rather than . (a) Haematocrit bluntness index, (b) Velocity bluntness index. Error bars show one standard deviation. (TIFF) [file pone.0100473.s002.tiff]
