# Supplementary material for: Spatial Distributions of Red Blood Cells Significantly Alter Local Haemodynamics
Source: PLoS One. 2014 Jun 20;9(6):e100473. doi: 10.1371/journal.pone.0100473 (PMC4065105; doi:10.1371/journal.pone.0100473)
Supplement: Information S1 — Image pre-processing. Details of the image processing methods applied prior to PIV and image intensity analysis. (PDF) [file pone.0100473.s003.pdf]

Spatial distributions of red blood cells significantly alter local haemodynamics

Joseph M. Sherwood, David Holmes, Efstathios Kaliviotis, Stavroula Balabani

Supporting Information S1: Image pre-processing

The focussing of the microstrobe resulted in an uneven illumination across the image. This was corrected for by calculating the mean image for each case, masking out the channel and fitting a Gaussian distribution to the remaining image. The Gaussian fit was normalised such that it had a maximum of one. Each image in the case was then divided by the normalised Gaussian fit to remove the uneven illumination. Image orientation was corrected as follows. First, the standard deviation of each pixel across all of the images in a case was calculated. The resulting image highlighted the channel location within the image. The standard deviation image was repeatedly eroded to smooth the edges, and then skeletonised to provide a single line for each branch. The orientation of the main channel was used to calculate an angle by which to rotate the image using cubic spline interpolation to ensure the branches were orthogonal with the image dimensions. The images were then scaled using cubic interpolation from $0.649\mu m/px$ (77 pixels per channel width) to $0.595\mu m/px$ (84 pixels per channel width). Although this scaling process does not add information, the regions of blurring due to diffraction/refraction at the channel wall (see Figure 2) were found to be consistently 4 pixels after scaling, which left a usable image region of 76 pixels. This was necessarily a multiple of 4, so that the PIV interrogation windows could fit exactly within the image. Finally, the images were then trimmed to $16w \times 5.5w$ as can be seen in the sample image in Figure 2a.
