# Supplementary material for: Spatial Distributions of Red Blood Cells Significantly Alter Local Haemodynamics
Source: PLoS One. 2014 Jun 20;9(6):e100473. doi: 10.1371/journal.pone.0100473 (PMC4065105; doi:10.1371/journal.pone.0100473)
Supplement: Information S2 — Haematocrit calculations. Description of the method applied for calibrating the haematocrit-intensity relationship and application of the technique to the bifurcations. (PDF) [file pone.0100473.s004.pdf]

Spatial distributions of red blood cells significantly alter local haemodynamics

Joseph M. Sherwood, David Holmes, Efstathios Kaliviotis, Stavroula Balabani

Supporting Information S2: Haematocrit calculations

Haematocrit Calibration

To increase uniformity between image sets, the raw image intensity, I_{raw} was first normalised to give $0 < I < 1$. This was done by calculating the average value of a given region of the mean image outside of channel, to give a maximum intensity for the image, I_{max}. This value is equivalent to that calculated for a channel filled only with PBS. The images were then scaled by multiplication with $1/I_{max}$. Uncertainty in this calculation, dI_{max} was quantified according to the standard deviation of the pixel intensity over the region where I_{max} was calculated.

Calibration was carried out considering a range of feed haematocrits and flow rates. For each case, a region of 4 channel widths was extracted from the mean image. An intensity profile, $I(y^*)$ was calculated using the mean value of all images at each radial location. Uncertainty in the intensity profile at each radial location was defined using the standard error of the mean, $dI_{SEM}(y^*)$ (as the hypothesis is that the mean intensity is proportional to haematocrit). The final normalised intensity profile $I^*(y^*)$ was then defined according to

$$I^*(y^*) = 1 - I(y^*) = \left(1 - \frac{I_{raw}(y^*)}{I_{max}}\right) \tag{S1}$$

so that $I^*(y^*)$ was proportional to $H(y^*)$. An estimate of the uncertainty $dI^*(y^*)$ was calculated using the chain rule of differentiation

$$dI(y^*) = \sqrt{\left(\frac{dI_{SEM}(y^*)}{I_{max}}\right)^2 + \left(\frac{dI_{max} I_{raw}(y^*)}{I_{max}^2}\right)^2} \tag{S2}$$

As can be seen in Figure 2b, close to the channel walls diffraction/refraction results in a small region of low image intensity, even in the absence of RBCs, which encroaches on the channel. In order to account for this, a method similar to that reported previously (Sherwood et al., 2014) was used: the six pixels ($\approx 3.5\mu m$) closest to the wall were discarded, and replaced with values calculated by linearly extrapolating the next six pixels in the intensity profile $I^*(y^*)$. An equivalent operation was carried out to extrapolate $dI^*(y^*)$.

The normalised intensity profile must be related to the mean haematocrit for each case in order to derive the relationship between the two. The discharge haematocrit, H_D was estimated by correcting the feed haematocrit, H_F, empirically for red cell screening at the channel entrance according to the fits of Gaehtgens et al. (1978) for aggregating and non-aggregating samples

$$H_D = H_F(C + D\log U^*) \tag{S3}$$

The parameters C and D are 0.901 and 0.029 respectively for the aggregating (Dextran) samples and 0.878 and 0.042 respectively for the non-aggregating (PBS) samples. It should be noted that the hydraulic diameter for a square channel of aspect ratio unity is equal to the side length, w. Uncertainty in U^* was found to have negligible impact on the correction for RBC screening.

To account for the Fåhraeus effect, the empirical equation defined by Pries et al. (1990) was used to estimate the channel haematocrit, H_C

$$H_C = H_D\left(H_D + (1 - H_D)\left(1 + e^{-0.415w} - 0.6e^{-0.011w}\right)\right) \tag{S4}$$

Given the empirical nature of this correction, it is not possible to estimate a reasonable uncertainty. However, any inaccuracy will be systematic, and hence will affect all data sets similarly.

In order to convert normalised intensity profiles into haematocrit profiles, the desired relationship is

$$H\left(y^{*}\right) = f\left(I\left(y^{*}\right)\right) \tag{S5}$$

for which the function, f, must be derived. However, $H\left(y^{*}\right)$ is not available, rather the average channel haematocrit is known for each case, $\overline{H_C}$. The overbar notation is used to distinguish the mean from the local haematocrit, where an average quantity is defined as:

$$\overline{\phi} = \int_{-0.5}^{0.5} \phi\left(y^{*}\right) dy^{*} \tag{S6}$$

calculated using trapezoidal integration. It can then be assumed that if $\overline{H_C} = \overline{f\left(I^{*}\right)}$, then Equation S5 is also valid, and f can be used for calculating the haematocrit from the acquired images. The average normalised intensity, $\overline{I^{*}}$, is calculated according to Equation S6 with $\phi = I^{*}\left(y^{*}\right)$ and the uncertainty, $d\overline{I^{*}}$, is calculated using Equation S6 with $\phi = dI^{*}\left(y^{*}\right)$. However, unless f is a constant (i.e. the haematocrit/intensity relationship is linear), $\overline{f\left(I^{*}\right)} \neq f\left(\overline{I^{*}}\right)$, hence calculation of f becomes more difficult. In order to predict a form for f, $\overline{I^{*}}(\pm 1.96 d\overline{I^{*}}$ to give 95% confidence intervals) was plotted against $\overline{H_C}$ as shown by the points in Figure S1.

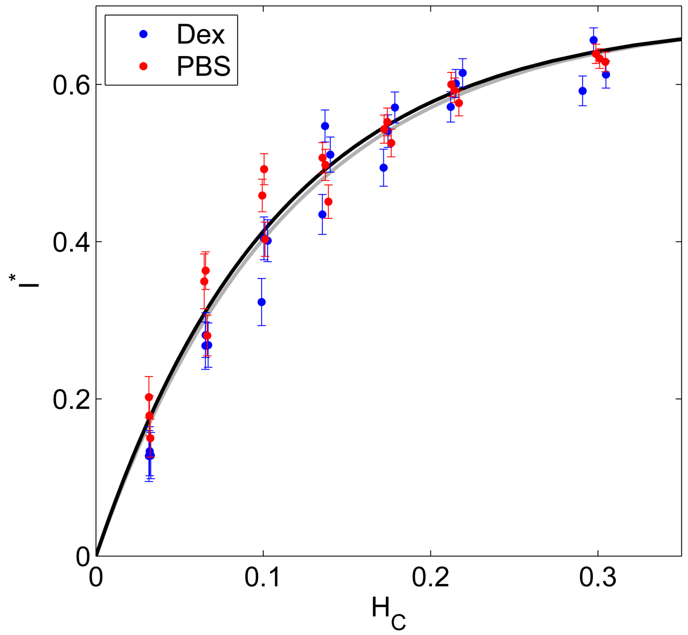

Figure S1: Haematocrit - intensity calibration. Result of the calibration, showing haematocrit against normalised image intensity. Dots show $\overline{I^{*}}$ against $\overline{H_C}$, with errorbars showing 1.96 standard deviations. Grey line shows best fit to Equation S7 based on non-linear regression. The black line shows fitted calibration curve after minimisation, $f\left(I^{*}\right)$ with parameters calculated as described in the text.

An exponential relationship of the form

$$I^{*} = a\left(1 - e^{-bH}\right) \tag{S7}$$

was identified, where a is the saturation point at which increasing haematocrit will not yield a darker image and b defines the non-linearity of the haematocrit-intensity relationship. Note that the overbar notation is not

adopted in Equation S7, as this represents a proposed form for f which should be applicable to local values. Rearranging Equation S7 to make H the subject:

$$H = f(I^*) = \frac{-1}{b} \ln\left(1 - \frac{I^*}{a}\right) \tag{S8}$$

yields a model for f for which a and b must be derived based on $I^*(y^*)$ and $\overline{H_C}$. By assuming that $\overline{f(I^*)} = f(\overline{I^*})$, an initial guess for the parameters a and b was made via fitting Equation S7 to the data using non-linear regression. This yields initial estimates of the fitting parameters, $a_0 = 0.689$ and $b_0 = 8.804$ respectively, which give the grey line in Figure S1.
An iterative search algorithm was then applied to minimise the error function

$$E = \sqrt{\frac{1}{n}\sum_{i=1}^{n}\left(\frac{\overline{H_{C_i}} - \overline{H_{E_i}}}{\overline{dH_{E_i}}}\right)^2} \tag{S9}$$

For each combination of a and b, the haematocrit profile, $H_E(y^*)$ was calculated according to Equation S8 and the uncertainty in the haematocrit profile was calculated according to

$$dH_E(y^*) = f(I^*(y^*) + dI^*(y^*)) - f(I^*(y^*)) \tag{S10}$$

due to the nonlinearity of f. $\overline{H_{E_i}}$ and $\overline{dH_{E_i}}$ were then calculated according to Equation S6. Starting at a_0 and b_0, a range of the fitting parameters a and b in increments of 0.1 was searched and the values which gave the smallest value of E were selected. The resolution of the search was reduced to 0.01 and the process repeated. A third iteration with a resolution of 0.001 yielded $a = 0.685$ and $b = 9.244$ to three decimal places. The calculated fit is shown by the black line in Figure S1. An estimate of the uncertainty in the fitting procedure is given by the weighted standard deviation of the residuals, yielding $\sigma_H = 0.014$. If the same parameter is calculated based on the initial estimates, a_0 and b_0, then $\sigma_{H_0} = 0.015$. Furthermore, if only the aggregating or non-aggregating samples are used to apply the fit, $\sigma_H = 0.017$ and $\sigma_H = 0.015$ respectively. Given that the residuals about the fit calculated by minimising the least square error should be normally distributed, a two-tailed t-test was used to compare the residuals calculated using the combined Dextran and PBS data sets to just the Dextran or PBS data sets. No significant difference was found ($p > 0.2$).
These comparisons indicate that the technique is robust and additionally, that in future applications of the technique, simple non-linear regression between $\overline{I^*}$ and $\overline{H_C}$ should be sufficient.

Although the methodologies differ, the present results are qualitatively comparable to the microphotometric approach of Pries et al. (1983), where I^* is analogous to the optical density. The relatively low saturation haematocrit and high nonlinearity, means that the methodology becomes insensitive for haematocrits greater than 0.3. However, as the approach is intended for application to channels on the scale of arterioles, wherein haematocrit is significantly lower than that in the large arteries, a haematocrit range limitation for the technique of $0 - 0.3$ is sufficient.

Haematocrit in the bifurcating microchannel

For the bifurcation images, the image illumination was adjusted to match that of the calibration images for the same feed haematocrit, to ensure that the calibration could be accurately applied. Firstly, the image histograms of the normalised calibration images and the normalised parent branch of the bifurcation images were calculated. Only the parent branch of the bifurcation image was used as this long straight section should have the same properties as the calibration images. The histogram of the entire bifurcation image was then adjusted by mapping the 0.1 and 99.9^{th} percentiles of the histograms in the parent branch to match those from the calibration image. This was carried out on each image and the mean corrected image was calculated.

The haematocrit in each branch was then calculated as follows. The corrected mean image, excluding the six pixels closest to each wall, was treated as described above for the calibration images. In order to smooth the data in the axial direction, at a given axial location i, the intensity profile was calculated from the mean intensity over a region from $i-6$ to $i+6$ ($7.7\mu m$). The uncertainty in the profile was calculated as described above, according to the standard error of the mean along the averaging region at each radial location. This yielded the $(x, y))$ resolved intensity distribution $I_s^*(x, y)) \pm dI_{s,SEM}^*(x, y)$. The smoothed intensity image was then converted via Equation S8 to yield $H(x, y)$. The uncertainty due the averaging $(dH_a(x, y))$ was calculated by applying Equation S10 to the intensity distribution. The uncertainty in the estimated haematocrit was then given by combining the averaging and fitting errors in quadrature:

$$dH(x, y) = \sqrt{(dH_a(x, y))^2 + \sigma_H^2} \tag{S11}$$

Details of the treatment of these distributions are provided in the main text and Supporting information S3.

References

Gaehtgens, P., Kreutz, F. & Albrecht, K. (1978), "Fåhraeus effect and cell screening during tube flow of human blood. II. Effect of Dextran-induced cell aggregation", *Biorheology* **15**, 155–161.

Pries, A., Kanzow, G. & Gaehtgens, P. (1983), "Microphotometric determination of hematocrit in small vessels.", *American Journal of Physiology* **245(1)**, H167–177.

Pries, A., Secomb, T., Gaehtgens, P. & Gross, J. (1990), "Blood flow in microvascular networks - experiments and simulation", *Circulation Research* **67**, 826–834.

Sherwood, J., Dusting, J., Kaliviotis, E. & Balabani, S. (2014), "Hematocrit, Viscosity and Velocity Distributions of Aggregating and Non-Aggregating Blood in a Bifurcating Microchannel", *Biomechanics and Modeling in Mechanobiology* **13(2)**, 259–273.
