# Supplementary material for: Spatial Distributions of Red Blood Cells Significantly Alter Local Haemodynamics
Source: PLoS One. 2014 Jun 20;9(6):e100473. doi: 10.1371/journal.pone.0100473 (PMC4065105; doi:10.1371/journal.pone.0100473)
Supplement: Information S3 — Quasi-3D assumptions. Explanation of the mathematical formulation of the assumptions used to infer three-dimensional distributions of haematocrit and velocity. (PDF) [file pone.0100473.s005.pdf]

Spatial distributions of red blood cells significantly alter local haemodynamics

Joseph M. Sherwood, David Holmes, Efstathios Kaliviotis, Stavroula Balabani

Supporting Information S3: Quasi-3D assumptions

Based on the concept that the parent branch represents an equilibrium condition, the *yz*-plane distribution of a parameter in the parent branch (which could be velocity or haematocrit) should have 4-fold rotational symmetry, i.e.

$$\psi_p\left(x_p^*, -y_i^*, 0\right) = \psi_p\left(x_p^*, y_i^*, 0\right) = \psi_p\left(x_p^*, 0, -y_i^*\right) = \psi_p\left(x_p^*, 0, y_i^*\right) \tag{S12}$$

Where x_p^* is the x^* location at the centre of the parent branch ROI and y_i^* is an arbitrary co-ordinate between -0.5 and 0.5. The haematocrit distribution and PIV methodologies yield parent branch profiles in the *xy* plane that can be used to estimate the full *yz* plane distribution. Profiles in this section are defined by averaging over the ROI indicated in Figure 2a.

Haematocrit

For the haematocrit distribution, wherein each value in the profile represents the average along the *z*-axis at that location:

$$H_p\left(x_p^*, y_i^*, z_i^*\right) = H_p\left(x_p^*, y_i^*, 0\right)\frac{H_p\left(x_p^*, z_i^*, 0\right)}{\overline{H_p}} \tag{S13}$$

For $-0.5 \leq y_i^* \leq 0.5$ and $-0.5 \leq z_i^* \leq 0.5$. In practice, the measured haematocrit profiles have a certain degree of noise and asymmetry. In order to avoid propagating these irregularities into the *z*-axis, the parent branch data are first fitted to a profile, derived from the form of the analytical solution for the velocity in a square microchannel, with simplifications and additions for non-zero wall haematocrit and varying bluntness:

$$H_{sym} = h\left(\frac{\cosh\left(0.5^m\right) - \cosh\left(\left|y_i^*\right|^m\right)}{\cosh\left(0.5^m\right) - 1}\right) + h_0 \tag{S14}$$

where h is a weighting parameter related to the average haematocrit, h_0 is the haematocrit at the wall and the exponent m defines the bluntness of the haematocrit profile. The haematocrit profile $H_p \pm dH_p$ in the parent branch ROI was mirrored about the $x^* = 0$ line and a symmetric profile was calculated from the weighted mean at each y^* location. The uncertainty in the profile was given by the weighted standard deviation. This profile was then fit to Equation S14 using robust weighted non-linear regression in Matlab to give $H_{sym} \pm dH_{sym}$. The weights were defined by $(\frac{1}{dH_p^2})$ and dH_{sym} was calculated as the 68% confidence intervals from the fit. The average root mean square difference between the measured values and the fitted profiles were 5.2% and 3.6% of the average haematocrit for the Dextran and PBS cases respectively.

As the geometry of the microchannel is planar, it can be assumed that the shape of the *z*-axis distribution (H_{sym}) does not change throughout the domain, hence, for branch j

$$H_j\left(x_j^*, y_i^*, z_i^*\right) = H_j\left(x_j^*, y_i^*, 0\right)\frac{H_{sym}\left(x_p^*, z_i^*, 0\right)}{\overline{H_p}} \tag{S15}$$

for the parent, middle and outlet branches, and with the appropriate co-ordinate changes (x^* and y^* are swapped) in the daughter branches. The uncertainty in H_j is given by the chain rule of differentiation

$$dH_j = \frac{1}{\overline{H_p}}\sqrt{\left(dH_j.H_{sym}\right)^2 + \left(H_j.dH_{sym}\right)^2 + \left(\frac{\overline{dH_p}.H_j.H_{sym}}{\overline{H_p}}\right)^2} \tag{S16}$$

where the co-ordinates have been omitted from Equation S16 for clarity and $\overline{dH_p}$ is calculated by integrating dH_p across the channel.

Velocity

For velocity, a similar logic can be used, however the measured profile ideally represents the maximum velocity in the z-axis, $V_{\max}$ (defined based on the symmetric profile), rather than the mean (as was the case for haematocrit). Hence, the velocity profile in the parent branch is fit to

$$V_{sym} = v\left(\frac{\cosh\left(0.5^m\right) - \cosh\left(\left|y_i^*\right|^m\right)}{\cosh\left(0.5^m\right) - 1}\right) + v_0 \tag{S17}$$

where v is a weighting parameter, related to the average velocity, v_0 is the slip velocity and the exponent m defines the bluntness of the velocity profile. The average root mean square difference between the measured values and the fitted profiles (a combination of errors from forcing symmetry and the fitting algorithm) were 0.9% and 2.0% of the average of velocity profile for the Dextran and PBS cases respectively.

The yz-plane distributions are calculated according to

$$V_j\left(x_j^*, y_i^*, z_i^*\right) = V_j\left(x_j^*, y_i^*, 0\right)\frac{V_p\left(x_p^*, z_i^*, 0\right)}{V_{\max}} \tag{S18}$$

The uncertainty in V_j is given by

$$dV_j = \frac{1}{V_{\max}}\sqrt{\left(dV_j.V_{sym}\right)^2 + \left(V_j.dV_{sym}\right)^2 + \left(\frac{dV_{\max}.V_j.V_{sym}}{V_{\max}}\right)^2} \tag{S19}$$

With $dV_{\max}$ calculated as dV_{sym} evaluated at the location of $V_{\max}$.
