# Supplementary material for: Spatial Distributions of Red Blood Cells Significantly Alter Local Haemodynamics
Source: PLoS One. 2014 Jun 20;9(6):e100473. doi: 10.1371/journal.pone.0100473 (PMC4065105; doi:10.1371/journal.pone.0100473)
Supplement: Information S4 — Volumetric and RBC mass flow rate. Description of the calculations of flow rate parameters from the acquired data and analysis of deviations from continuity. (PDF) [file pone.0100473.s006.pdf]

Spatial distributions of red blood cells significantly alter local haemodynamics

Joseph M. Sherwood, David Holmes, Efstathios Kaliviotis, Stavroula Balabani

Supporting Information S4: Volumetric and RBC mass flow rate

The flow rate in each branch is calculated by integrating the yz-plane velocity distribution at location x_j:

$$Q_j = \int_{-0.5}^{0.5} \int_{-0.5}^{0.5} V_j \left(x_j^*, y^*, z^* \right) dy^* dz^* \tag{S20}$$

The uncertainty in the flow rate is given by

$$dQ_j = \int_{-0.5}^{0.5} \int_{-0.5}^{0.5} dV_j \left(x_j^*, y^*, z^* \right) dy^* dz^* \tag{S21}$$

The RBC mass flow rate across the yz-plane in the ROI of branch j can be calculated according to

$$F_j = \rho \int_{-0.5}^{0.5} \int_{-0.5}^{0.5} V_j \left(x_j^*, y^*, z^* \right) H_j \left(x_j^*, y^*, z^* \right) dy^* dz^* \tag{S22}$$

where ρ is the density of the RBC. The uncertainty in the RBC flux is given by

$$dF_j = \rho \int_{-0.5}^{0.5} \int_{-0.5}^{0.5} \sqrt{\left(dV_j \left(x_j^*, y^*, z^* \right) H_j \left(x_j^*, y^*, z^* \right) \right)^2 + \left(V_j \left(x_j^*, y^*, z^* \right) dH_j \left(x_j^*, y^*, z^* \right) \right)^2} dy^* dz^* \tag{S23}$$

Both volumetric and mass flux must be conserved throughout the domain, and deviations from this behaviour provide an indication in measurement error of the described techniques. An estimate for the error in the mass flux calculation in the first bifurcation can be determined according to

$$e_1 = \frac{F_p - F_{d1} - F_m}{F_p} \tag{S24}$$

where the subscripts $d1$, m and p refer to daughter branch 1, the middle branch and the parent branch respectively. In the second bifurcation,

$$e_2 = \frac{F_m - F_{d2} - F_o}{F_m} \tag{S25}$$

where the subscripts $d2$ and o refer to daughter branch 2 and the outlet branch respectively. For the whole microchannel

$$e_m = \frac{F_p - F_{d1} - F_{d2} - F_o}{F_p} \tag{S26}$$

Uncertainties in error in the flux calculations are estimated using the chain rule of differentiation. Equivalent calculations can be carried out for the volumetric flux, replacing F with Q in Equations S24-S26. Values of the error in the flux calculations are provided in Table 2.
